# A nationwide survey of anthelmintic treatment failure on sheep farms in Ireland

**DOI:** 10.1186/s13620-017-0086-9

**Published:** 2017-02-09

**Authors:** Jason D. Keegan, Orla M. Keane, Barbara Good, Theo De Waal, Marian Denny, James P. Hanrahan, William Fitzgerald, Maresa Sheehan

**Affiliations:** 1 Animal & Bioscience Department, Teagasc Grange, Dunsany, Co., Meath, Ireland; 2 Department of Agriculture, Food and the Marine, Central Veterinary Research Laboratory, Backweston, Co., Kildare, Ireland; 3 School of Veterinary Medicine, University College Dublin, Belfield, Dublin 4, Ireland; 4 Animal & Grassland Research & Innovation Centre, Teagasc, Athenry, Co., Galway, Ireland; 5 Kilkenny Regional Veterinary Laboratory, Hebron Road, Kilkenny, Co., Kilkenny, Ireland; 6 Limerick Regional Veterinary Laboratory, Knockalisheen, Co., Limerick, Ireland

## Abstract

**Background:**

Between 2013 and 2015 the Department of Agriculture, Food and the Marine (DAFM) administered a sheep technology adoption programme (STAP), with the aim of increasing profitability on Irish sheep farms by encouraging the adoption of best management practices. One of the options available to STAP participants was to test the efficacy of the anthelmintic treatment (benzimadazole, levamisole or macrocyclic lactone) used in their flocks by means of a drench test, which is a modification of the faecal egg count reduction test; individual faecal samples were collected from the same group of lambs before and after anthelmintic treatment, the number of eggs present pre and post treatment was subsequently determined from a pooled sample.

**Results:**

In total, 4211 drench tests were undertaken by farmers during the 3 years of the programme. Information on the anthelmintic product used was available for 3771 of these tests; anthelmintics from the classes benzimidazole (BZ), levamisole (LV) and macrocyclic lactone (ML) (avermectins (AVM) plus moxidectin (MOX)) were used in 42.0%, 23.4% and 32.5% of tests, respectively. The remaining 2.1% of tests involved an inappropriate product. The efficacy of treatment against ‘other trichostrongyles’ (excluding *Nematodirus* spp and *Strongyloides papillosus*.) could be established for 1446 tests, and 51% of these tests were considered effective (i.e. a reduction of faecal egg count (FEC) ≥ 95%). There was a significant difference among the drug groups in efficacy; 31.5%, 51.9%, 62.5% and 84% of treatments were considered effective for BZ, LV, AVM, MOX, respectively. The efficacy of treatment against *Nematodirus* spp. could be established for 338 tests and the overall efficacy was 96%.

**Conclusions:**

Due to the significant difference among the anthelmintic classes for efficacy against ‘other trichostrongyles’ along with the high level of efficacy against *Nematodirus* spp., a genus for which anthelmintic resistance is rarely reported, it is concluded that anthelmintic resistance was responsible for the majority of the anthelmintic treatment failures observed.

## Background

In grass-based, sheep-production systems, the administration of broad spectrum anthelmintic products is an important part of most strategies for the control of gastrointestinal nematodes (GIN). In Ireland, there are 5 classes of anthelmintic available for use in the control of GIN infections in sheep. Two of these products, an amino-acetonitrile derivative (AAD) and a spiroindole/macrocyclic lactone combination (SI) are currently only available on prescription and, therefore, are rarely used. The remaining anthelmintic classes commonly used to control sheep GIN in Ireland are benzimidazole (BZ), levamisole (LV) and macrocyclic lactone (ML). Anthelmintic resistance has become commonplace in many parts of the world and reports of multiple drug resistant nematodes are also increasing [1–5]. The development of anthelmintic resistant nematodes threatens the utility of a chemoprophylactic approach to GIN control.

The most common mechanism utilised to detect anthelmintic resistance is the faecal egg count reduction test (FECRT) [6], and evidence for BZ and LV resistance was found on 88% and 39% of farms, respectively, in Ireland using this method. Additionally, 11% of farms surveyed were suspected to have ML resistance by means of the in vitro DrenchRite® assay [7]. In Northern Ireland, evidence for BZ, LV and ML resistant nematodes was found in 81%, 14% and 57% of flocks, respectively [8].

Evidence from a recent study of management systems on lowland sheep farms showed that there was considerable departure from best practice in the use of GIN control measures [9], which can accelerate the development of anthelmintic resistance. It is imperative that sheep producers know what products are effective on their holdings and be aware of the improvements/adaptations that need to be made to their GIN control strategies in order to reduce the selective pressure for anthelmintic resistance. A sheep technology adoption programme (STAP), designed to increase profitability on Irish sheep farms by encouraging the adoption of ‘best management practices’, was established in 2013 [10]. As part of the programme, producers were required to complete two technical tasks from a list of 10. Among the list of STAP tasks was a “drench test” designed to evaluate the efficacy of their anthelmintic treatment by performing a faecal egg count on a composite faecal sample pre- and post-anthelmintic treatment. Preliminary results from the first year of this programme have been reported [11]. In this paper we report the drench test efficacy data from all three years of the programme.

## Methods

### Farm profile

In order to qualify for inclusion in STAP, producers were required to have a minimum of 30 breeding ewes or to have purchased a minimum of 100 lambs/hoggets for breeding within the previous 2 years. Sheep Technology Adoption Programme participants who were selling lambs to processors were also required to apply for membership of the Board Bia Lamb Quality Assurance Scheme [12].

### Sample collection

In 2013, farmers who chose to carry out the drench test task were given a detailed set of instructions describing the sampling protocol [10]. Changes were made to the instructions and submission forms, following the response in 2013, to make them clearer and easier to complete [13, 14]. Instructions were issued to sheep producers by discussion-group facilitators and were also available for download from the DAFM website. It was stipulated that the drench test should be carried out post-weaning, on lambs that had not been treated with an anthelmintic product in the previous 6 weeks. In order to conduct the drench test, it was advised that between 15 and 20 lambs were placed in a clean pen and left for a period of time to allow them to defecate. A minimum of 10 fresh faecal deposits (representing different lambs) were to be collected and each placed in a separate transport container and to be sent by mail to a DAFM approved testing laboratory as soon as possible after collection. Farmers were instructed to: ((1) mark the group of lambs that were to be sampled; (2) treat the lambs with an anthelmintic product of their choice from the BZ, LV or ML classes of anthelmintic; weigh the 3 largest lambs in the group and treat to the weight of the heaviest lamb in accordance with the manufacturer’s recommendations; (4) calibrate the dosing gun before use and to ensure that the anthelmintic product was in date and well mixed before administration. (5) Ten fresh faecal samples were to be collected post-treatment from the same group of marked lambs and sent to the DAFM approved lab for testing. It was advised to keep samples refrigerated if it was not possible to post them on the day of sampling; Post-treatment samples were to be taken seven days post-treatment if a LV product was used, or 14 days post-treatment if a BZ or ML product was used, in line with World Association for the Advancement of Veterinary Parasitology (WAAVP) guidelines [6].

### Faecal egg count

Eight commercial laboratories were approved by DAFM to test faecal samples. In order for a laboratory to be considered for approval, participation in proficiency testing was required. The proficiency tests were administered by Vetqas, the independent accredited proficiency testing unit of the United Kingdom’s Animal and Plant Health Agency (APHA). The STAP drench test consisted of a faecal egg count carried out on a composite faecal sample. Laboratories were provided with a protocol detailing how to generate the composite samples and how to enumerate the eggs in faeces. Briefly, for each group of lambs to be tested, composite faecal samples were prepared so that each individual animal sample contributed the same unit weight to the composite sample (3 g per individual sample). Faecal egg counts were carried out according to the standard McMaster method with a sensitivity of 50 eggs per gram (EPG) of faeces [15]. Eggs of *Nematodirus* spp. (FEC_NEM_) and the other trichostrongyles (FEC_OT_) (excluding Nematodirus spp and *Strongyloides papillosus*) were enumerated separately.

### Data management and statistical analysis

Data were entered in an Excel spreadsheet and checked for anomalies. Subsequent editing was applied to exclude data in which: (1) both a pre- and post-treatment sample were not provided; (2) the anthelmintic class used was not listed; (3) an ineligible product was used (i.e., SI, AAD or products only active as flukicides); (4) the sampling dates were not provided; (5) an incorrect sampling interval was used - data were included only if the sampling interval was 10 to 14, 4 to 7 or 14 to 18 days for BZ, LV and ML products, respectively; (6) the pre-treatment flock egg count was < 200 EPG (7) a proficiency test was not conducted, or failed, by the laboratory that carried out the test. Combination roundworm and fluke treatments were included within the BZ, LV and ML classes based on the active ingredients present while combination BZ/LV products were excluded.

The percentage of farmers choosing each anthelmintic class (i.e. BZ, LV and ML) over the 3 years of the study was examined using contingency tables. The reduction in faecal egg count was calculated as the ((Pre-treatment count - Post-treatment count)/Pre-treatment count)*100. Reductions for FEC_OT_ and FEC_NEM_ were calculated separately. The treatment was considered effective if the reduction in FEC was ≥ 95%. As the reduction in FEC was based on pooled faecal samples, a confidence interval was not calculated.

As selection for anthelmintic resistance in macrocyclic lactones appears to be different and moxidectin (MOX) resistance is less frequently reported than for avermectins (AVM) [2, 16–19] , these data were examined separately. Tests conducted in April (*n* = 1) and May (*n* = 4) were excluded. A further 7 tests were excluded as information was missing regarding which subgroup of ML (AVM or MOX) was used (*n* = 6) or where the farm was located (*n* = 1). Drench test efficacy against the other trichostrongyles was analysed for the remaining tests (*n* = 1434) using Proc GENMOD (SAS v9.1) with a logit link function to fit a model that had effects for year (2013, 2014, 2015), month (June, July, August, September), year*month, geographical region [Border, Mid-East, Midlands, South-East, South-West, West; referred to as NUTS (http://ec.europa.eu/eurostat/web/nuts/overview)] year*NUTS, anthelmintic group (BZ, LV, AVM, MOX), year*anthelmintic group and month*anthelmintic group. Due to low numbers of drench test results from the Dublin (*n* = 11) and the Mid-West regions (*n* = 4) data for these regions were included in the Mid-East and the South West regions, respectively.

## Results

### Choice of anthelmintic

More than 4200 drench tests were carried out by farmers over the 3 years of the programme. The anthelmintic product used was reported for 3771 of these tests. Benzimdazole was used in 42% of cases, making it the most popular anthelmintic class overall, followed by the ML class (32.5% of cases). Levamisole was the least popular class, being used in 23.4% of cases. A combination BZ/LV product, an SI or an AAD product, or a product only active as a flukicide was used in the remaining 2.1% (*n* = 78) of tests.

**Fig. 1:**
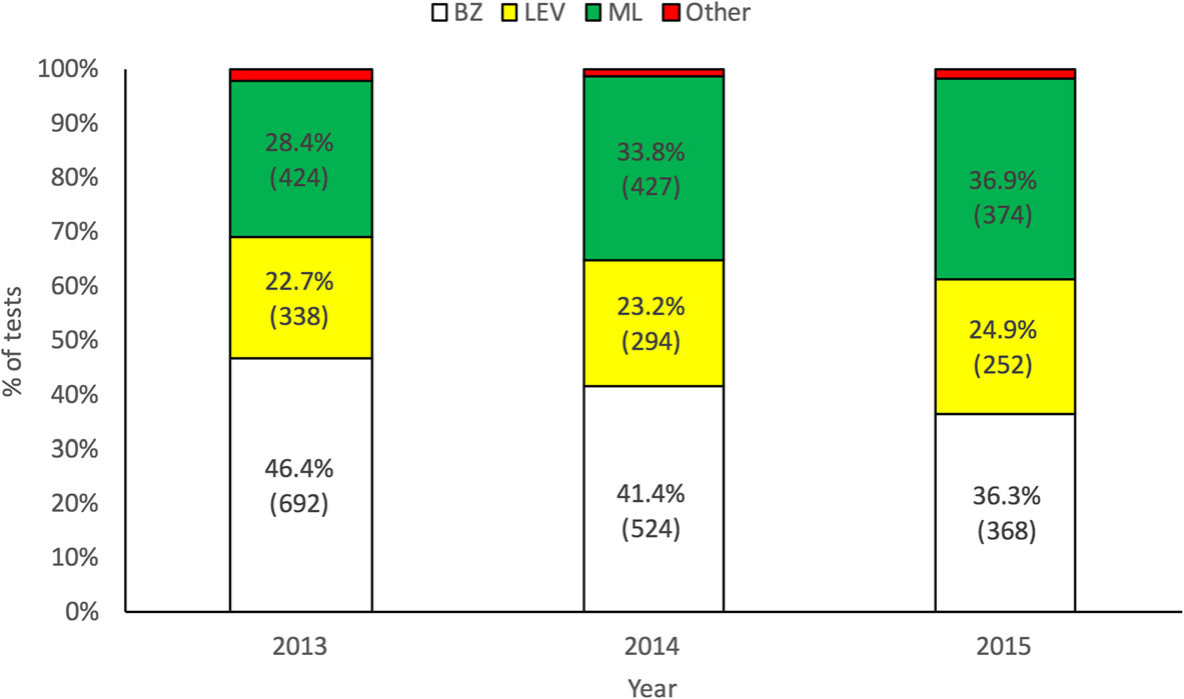
Anthelmintic classes used in drench tests for the years 2013 to 2015. Anthelmintic classes (BZ = benzimidazole; LV = levamisole; ML = macrocyclic lactone) used in drench tests in 2013 (*n* = 1492), 2014 (*n* = 1265), and 2015 (*n* = 1014). The number of tests is given in parentheses. Invalid tests (shown in red) were: 2.5% (38), 1.6% (20) and 2% (20) in 2013, 2014 and 2015, respectively

There was a significant difference in the number of drench tests carried out using BZ, LV, ML or an inappropriate product over the 3 years the study was conducted (χ^2^ = 33.47, *P* < 0.001). The percentage of drench tests carried out using LV was similar in each year, representing 22.7%, 23.2% and 24.9% of the tests carried out in 2013, 2014 and 2015 respectively. Similarly, the number of inappropriate treatments in each year was relatively consistent (2.5%, 1.6% and 2% in 2013, 2014 and 2015, respectively). The percentage of producers using BZ products declined from 46.4% in 2013 to 36.3% in 2015. Conversely, the percentage of farmers using ML products increased over the course of the 3 years from 28.4% in 2013 to 36.9% in 2015 (Fig. 1).

### Farmer participation and compliance with instructions

The level of participation and the number of drench tests that were removed through application of quality control procedures are shown for each year in Table 1. The greatest number of drench tests undertaken by producers was in 2013, with fewer participants taking part in subsequent years. However, only 27.8% of the drench tests carried out in 2013 were conducted according to the instructions provided. A greater level of compliance was observed in 2014 and 2015 (61.6% and 68.4% of tests conducted as instructed, respectively). Failure to adhere to the correct sampling interval for the anthelmintic class being tested was a major source of data loss in all years. Pretreatment FEC_OT_ or FEC_NEM_ was less than 200 EPG in 532 and 1681 tests, respectively, and these were not included in the analyses.

**Table 1:** Response to the drench test task

Item^a^	Year			All years
	2013	2014	2015	
Total number of participants:	1893(100)^b^	1279 (100)	1039 (100)	4211(100)
Non compliant /Missing data:	1366 (72.2)	491 (38.4)	328 (31.6)	2158 (51.8)
• Only one faecal sample submitted	308	24	54	386
• Ineligible product used	32	20	20	72
• Sampling date missing	130	4	5	139
• Incorrect sampling interval	619	432	233	1284
• Ineligible product used	32	20	20	72
• No product information	277	11	16	304
Complied with instructions:	527 (27.8)	788 (61.6)	711 (68.4)	2026(48.1)
• Initial FEC_OT_ < 200 epg	143	263	126	532
• Lab failed proficiency test	15	21	12	48
• Useable data for analysis of FEC_OT_	369	504	573	1446
• Initial FEC_NEM_ < 200 epg	410	677	594	1681
• Lab failed proficiency test	2	4	1	7
• Useable data for analysis of FEC_NEM_	115	107	116	338

^a^epg = eggs per gram; FEC_OT_ = faecal egg count for other trichostrongyles; FEC_NEM_= faecal egg count for Nematodirus spp.

^b^Percent in parentheses

### Effectiveness of anthelmintic treatment against ‘other trichostrongyles’

**Fig. 2:**
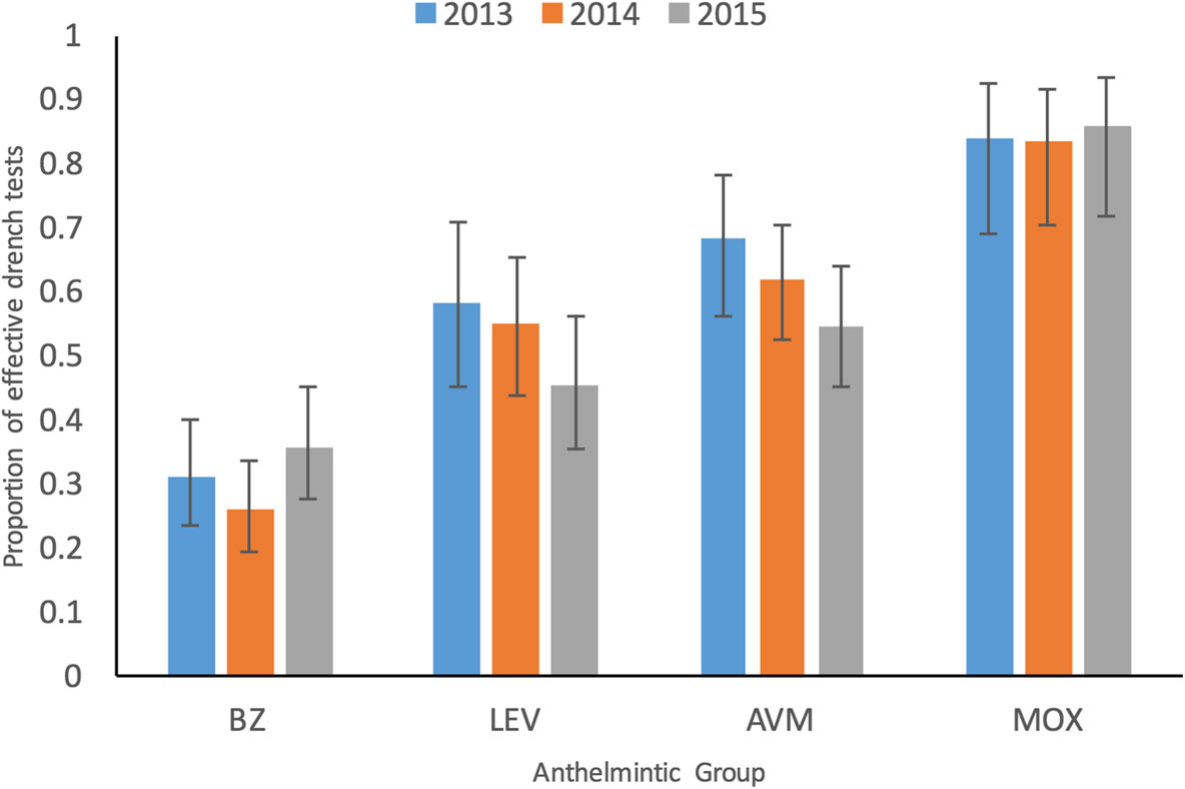
Anthelmintic effectiveness against ‘other trichostrongyles’ in each year. Estimates of the proportion (back-transformed least squares means) of anthelmintic treatments that were effective against ‘other trichostrongyles’ for each anthelmintic group (BZ = benzimadole; LV = levamisole; AVM = avermectin; MOX = moxidectin). Vertical bars represent 95% confidence interval

Overall, 51% of the treatments (*n* = 1446) were considered effective with similar levels of overall efficacy observed in each year (51%, 52% and 49% in 2013, 2014 and 2015, respectively). The proportion of treatments that was effective against ‘other trichostrongyles’ for each anthelmintic group in each year is shown in Fig. 2. There was no effect of year, month or geographical region on the efficacy of anthelmintic treatment. Differences in efficacy among the 4 anthelmintic groups were highly significant (χ^2^ = 128.92, *P* < 0.001). Benzimidazole treatments were the least effective in every year; only 31.5% of the total of 550 BZ treatments resulted in a reduction in FEC_OT_ of ≥ 95% while BZ effected no reduction in 16% of cases. Of the 316 tests using LV, 51.9% were considered effective and LV was the second least effective anthelmintic in each year. No reduction in FEC_OT_ was observed in 9% of tests involving LV. Avermectin was the second most effective treatment; 62.5% of 405 treatments resulted in a reduction ≥ 95% while only 5% resulted in no reduction in FEC_OT_. Moxidectin was the most effective anthelmintic, effective in 84% of treatments (*n* = 163) with only 3% of treatments resulting in no reduction in FEC_OT_.

**Fig. 3:**
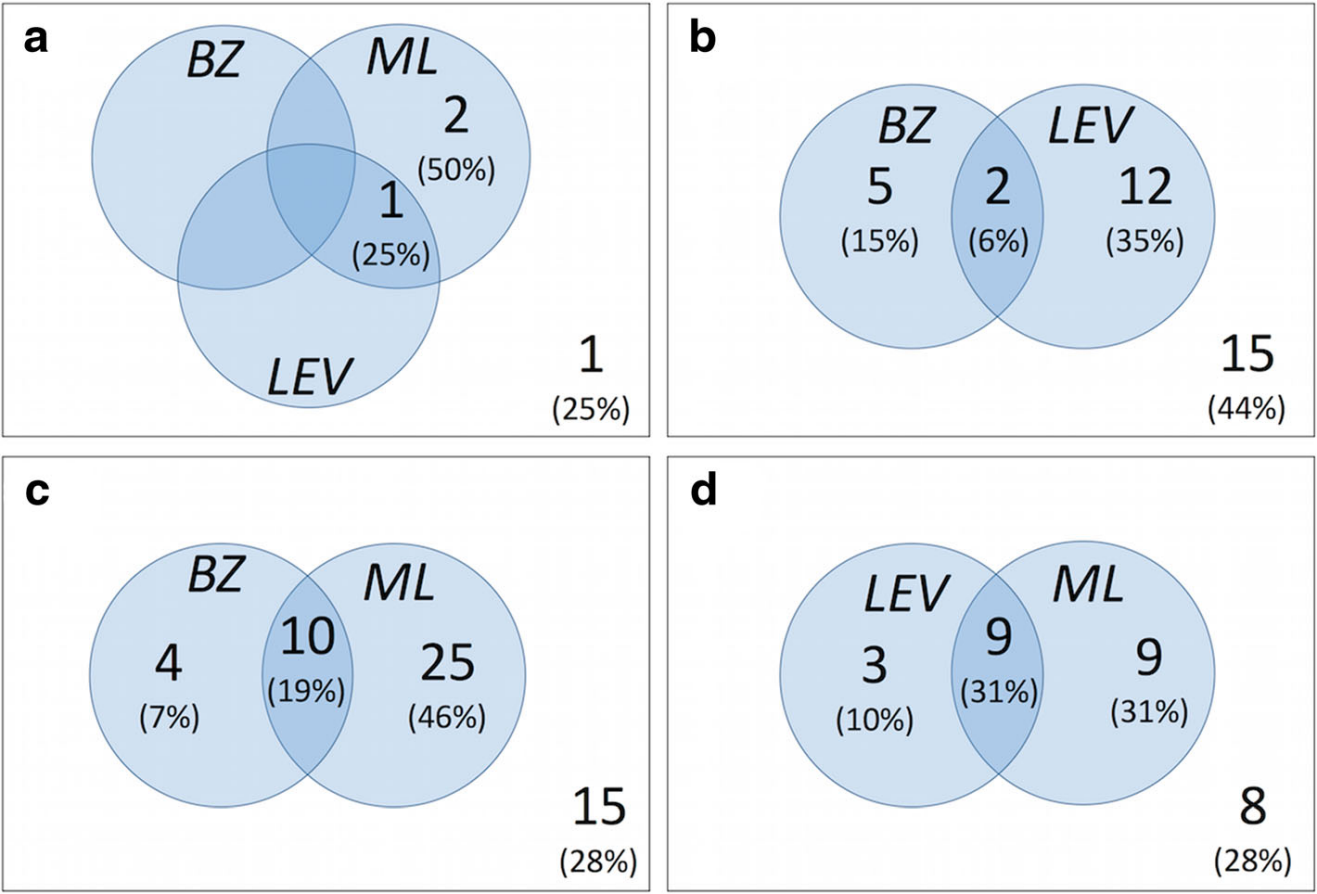
Multiple anthelmintic classes tested on the same farm. Venn diagram summary of results from drench tests on the same farm and involving more than 1 anthelmintic class (BZ = benzimidazole; LV = levamisole; ML = macrocyclic lactone). The number (%) of effective tests for each anthelmintic group is given in the corresponding set; set intersections show cases where multiple anthelmintics were effective. The numbers outside the sets represent the number of farms on which multiple anthelmintics were ineffective. **a** farms (*n* = 4) that provided results for BZ, LV and ML; **b** farms (*n* = 34) that provided test results for BZ and LV; **c** farms (*n* = 54) that provided test results for BZ and ML; **d** farms (*n* = 29) that provided results for LV and ML

A total of 121 producers carried out more than one drench test on the same farm using different anthelmintic classes (Fig. 3). There were only a small number of producers (*n* = 4) who tested all 3 anthelmintic classes, of which one farm demonstrated inefficacy of all 3 classes. Inefficacy of BZ plus LV (*n* = 34), BZ plus ML (*n* = 54) and LV plus ML (*n* = 29) was observed on 44%, 28% and 28% of farms, respectively.

### Effectiveness of anthelmintic treatment against *Nematodirus spp*.

A total of 338 drench tests met the criteria for calculating a reduction in FEC_NEM_; only 14 (4%) were considered ineffective (Table 2). Benzimidazole and LV inefficacy was observed in 5 cases each, while AVM and MOX inefficacy was observed in 2 cases each.

**Table 2:** Tests ineffective against *Nematodirus* spp.

Anthelmintic group	*Nematodirus* spp.			Other trichostrongyles			Year	Month	Location
	Pre-treatment FEC	Post-treatment FEC	Efficacy	Pre-treatment FEC	Post-treatment FEC	Efficacy^a^			
BZ	300	250	-	300	550	-	2014	July	Border
BZ	450	50	-	250	100	-	2014	June	South-West
BZ	200	550	-	1750	550	-	2015	June	South-East
BZ	2100	200	-	800	100	-	2015	June	West
BZ	300	50	-	600	950	-	2015	June	West
LV	300	100	-	700	1000	-	2013	July	Border
LV	3800	1200	-	800	100	-	2013	June	Mid-East
LV	450	3200	-	0	0	n/a	2013	September	Border
LV	3100	500	-	1600	0	+	2013	September	South-West
LV	300	50	-	150	0	+	2015	September	Midlands
AVM	1400	200	-	1000	0	+	2013	July	Mid-East
AVM	1350	1350	-	650	650	-	2015	June	Mid-East
MOX	850	250	-	350	0	+	2013	June	Mid-East
MOX	500	100	-	250	0	+	2013	June	Midlands

^a^+ = effective (reduction in FEC ≥ 95%); - = Not effective (reduction in FEC < 95%); n/a = invalid test

### Simultaneous efficacy against Nematodirus and ‘other trichostrongyles’

In total, 301 tests satisfied the criteria for calculation of reduction for both FEC_OT_ and FEC_NEM_. Tests that did not result in a reduction of 100% for FEC_NEM_ (*n* = 14) were excluded. The remaining tests represent cases in which an effective anthelmintic treatment was administered for *Nematodirus* spp. and, thus, any inefficacy against ‘other trichostrongyles’ is unlikely to be attributable to inappropriate dosing technique; consequently indicating anthelmintic resistance. The efficacy of treatment with each of the anthelmintic groups for each year is shown in Table 3. The resulting overall efficacy estimates were 30%, 56%, 69% and 97% for BZ, LV, AVM and MOX, respectively.

**Table 3:** Efficacy against other trichostrongyles for those treatments that were effective against Nematodirus, by year and anthelmintic

Anthelmintic	Tests	2013		2014		2015		All years	
		Efficacy		Efficacy		Efficacy		Efficacy	
		+	-	+	-	+	-	+	-
BZ	n	15	29	10	28	9	21	34	78
	*%*	*34*	*66*	*26*	*73*	*30*	*70*	*30*	*70*
LV	n	8	6	12	5	11	13	31	24
	*%*	*57*	*42*	*71*	*29*	*46*	*54*	*56*	*43*
AVR	n	20	6	20	6	23	16	63	28
	*%*	*77*	*23*	*77*	*23*	*59*	*41*	*69*	*31*
MOX	n	11	1	10	0	7	0	28	1
	*%*	*92*	*8*	*100*	*0*	*100*	*0*	*97*	*3*

+ effective (reduction in FEC ≥ 95%)

- not effective (reduction in FEC < 95%)

## Discussion

Approximately 10% of sheep producers in Ireland [20] participated in the STAP drench test task. To the authors’ knowledge, this is the largest field survey investigating anthelmintic inefficacy in the world to date and provides some baseline valuable data on treatment failure in Irish flocks. While the FECRT [6] is still regarded as the method of choice to investigate anthelmintic efficacy, the drench test employed in this survey represents a cost-effective modified version where samples from each flock were pooled instead of analysed separately by the laboratory, the results of which should be interpreted as an indicator of anthelmintic treatment failure and not anthelmintic resistance *per se* [21].

The majority of drench tests were carried out in the border and west regions, representing the areas of Ireland where the majority of sheep farming is conducted [20]. In the first year only 28% of the drench tests were carried out according to the instructions provided. Compliance improved in subsequent years following the redesign of the instructions, focused efforts by agricultural advisors to explain the task to farmers at meetings/farming press articles, and stricter enforcement of financial penalties for those not complying with the instructions. The high attrition rate (52% of samples), while clearly due to multiple factors such as those described earlier (Table 1), highlights the difficulties in establishing that protocols are correctly followed.

The reduction over time, in the proportion of farmers choosing a BZ product to test may reflect the increased awareness of the poor efficacy of BZ as a result of the publicity the programme received and reviews of results at discussion group meetings. As the proportion of producers using LV products remained relatively consistent, the decline in BZ use was reflected in an increase in the number of producers opting for ML products. In a previous study, the main factors influencing anthelmintic product choice by Irish sheep producers were their past experience with a particular anthelmintic and advice from their veterinary practitioner or agricultural adviser [9]. As the drench test task did not require producers to provide reasons for their choice of anthelmintic, it is not possible to say whether results of the task in preceding years influenced the anthelmintic choice of sheep farmers in subsequent years.

Of interest, the flock FEC pre-treatment was < 200 EPG in over a quarter of cases (who complied with the instructions), which is indicative of a low level of GIN parasitism. If the drench test was carried out at least 6 weeks after the last anthelmintic treatment (as per instructions), this suggests a large proportion of farmers may be treating unnecessarily. In a previous survey 86% of Irish sheep producers reported treating their animals according to a set programme [9]. This practice and the observation that 26% of anthelmintic treatments were administered to minimally parasitised animals reflect a departure from best practice GIN management. It also demonstrates the relevance of FEC as a management tool in informing the most appropriate time to treat as a reduction in the overall number of anthelmintic treatments administered has been observed when FEC is used to optimise the timing of anthelmintic treatments [22]. In the current programme, producers were requested to treat the animals directly after taking the first samples; in future studies the proviso to withhold treatment until the flock FEC is at least 200 EPG [21] should be included in the design. As well as ensuring a representative number of animals are included in the composite sample, FEC methods with greater analytical sensitivity (e.g. 15 EPG) [23, 24] would improve the diagnostic accuracy in future studies.

Despite being the most common anthelmintic class used by sheep producers, BZ treatment was found to be effective in only 31.5% of cases, with no reduction in FEC observed in 16% of cases. Drench tests can only establish whether an anthelmintic treatment was effective or not; the reason(s) for treatment failure cannot be established. Reasons for treatment failure may include: administering the incorrect dose rate due to use of faulty equipment, poor dosing technique incorrect storage or use of product resulting in reduced efficacy and anthelmintic resistance.

The levels of treatment failure observed in the current study are in broad agreement with previous work carried out to establish the prevalence of anthelmintic resistance on Irish sheep farms, which indicated GIN were susceptible to BZ, LV and ML anthelmintics on 39%, 72% and 89% of farms surveyed, respectively [7]. A high level of treatment efficacy against *Nematodirus* spp. was observed for all anthelmintic classes and there have been few reports of anthelmintic resistance in *Nematodirus* spp. in the UK [25, 26] and no published reports for Ireland. In a number of cases, it was possible to calculate both the reduction in FEC_OT_ and FEC_NEM_ from the same pooled faecal sample. Where treatment for one nematode group was 100% effective, it is most likely that an appropriate treatment was administered and that inappropriate dosing technique was not responsible for treatment failure. When only the tests that were 100% effective against *Nematodirus* spp. were taken into account, similar levels of efficacy were seen for each of the 4 anthelmintic groups as for the study as a whole, further suggesting that anthelmintic resistance was responsible for the failure of treatments to reduce the FEC for ‘other trichostrongyles’. Of the 14 tests that were considered ineffective against *Nematodirus* spp., 5 were considered effective against ‘other trichostrongyles’ suggesting that anthelmintic resistance could be responsible for the treatment failure in these cases. However further work would be required to confirm these findings.

More than one anthelmintic class was tested by some producers with inefficacy of multiple classes observed on some farms. Cases of multi-drug resistance in sheep nematodes have been reported in farms in Europe [1–5] and it is possible that in some cases where more than one anthelmintic failed, that multiple drug resistance is responsible. However, considering the small numbers involved, inappropriate treatment practices could also be responsible in some cases.

## Conclusions

The finding that almost half of all anthelmintic treatments administered to lambs were ineffective indicates that producers need to be encouraged to test the efficacy of anthelmintic treatments. Drench tests should be regarded as the first step to gaining a quick indicator of anthelmintic efficacy on farm and provide a catalyst for a more detailed exploration, e.g. FECRT, of whether anthelmintic resistance is indeed responsible if treatment failure is observed. Over 10% of the nation’s sheep producers were involved in performing a drench test as part of STAP. The interest generated by the publication of the results may encourage additional producers to test the effectiveness of their anthelmintic treatments. While the issue of the inefficacy of BZ against ‘other trichostrongyle’ populations in Ireland is not new [7], it is important that producers remain aware of the high level of efficacy of BZ as a targeted treatment against *Nematodirus* spp. There is also clear evidence to advocate the use of FEC as diagnostic support for decisions on anthelmintic administration. Strategies including the implementation of complementary control measures and the targeted use of prescription only anthelmintics to prolong the efficacy of other anthelmintics also need to be considered.

## Abbreviations

AAD: Amino-acetonitrile derivative
APHA: Animal and Plant Health Agency
AVM: Avermectin
BZ: Benzimidazole
DAFM: Department of Agriculture, Food and the Marine
EPG: Eggs per gram
FEC: Faecal egg count
FEC_NEM_: Faecal egg count for *Nematodirus* species
FEC_OT_: Faecal egg count for ‘other trichostrongyles’ (excluding *Strongyloides papillosus*)
FECRT: Faecal egg count reduction test
GIN: Gastrontestinal nematode
LV: Levamisole
ML: Macrocyclic lactone
MOX: Moxidectin
SI: Spiroindole
STAP: Sheep Technology Adoption Programme
WAAVP: World Association for the Advancement of Veterinary Parasitology
